# Lexical analysis of terms related to nursing care for pregnant women with syphilis

**DOI:** 10.1590/1980-220X-REEUSP-2025-0386en

**Published:** 2026-05-01

**Authors:** Ana Beatriz Pereira da Silva, Richardson Augusto Rosendo da Silva, Jocellem Alves de Medeiros, José Leonildo Fernandes de Queiroz, Vitória de Araújo e Silva, Amanda de Brito Rangel Pereira Sales, Jéssica Fernanda Galon, Elise Cristina dos Santos Felix

**Affiliations:** 1 Universidade Federal do Rio Grande do Norte, Programa de Pós-Graduação em Enfermagem, Natal, RN, Brazil.; 2 Universidade Federal do Rio Grande do Norte, Departamento de Enfermagem, Natal, RN, Brazil.; 3 Universidade de Ribeirão Preto, Departamento de Enfermagem, Ribeirão Preto, SP, Brazil.

**Keywords:** Syphilis, Pregnant People, Nursing Care, Prenatal Care, Standardized Nursing Terminology

## Abstract

**Objective:**

To analyze the lexicon related to nursing care for pregnant women with syphilis, based on the scientific literature.

**Method:**

A scoping review conducted according to the framework proposed by Arksey and O’Malley and the PRISMA-ScR guidelines. Searches were performed in national and international databases (PubMed, CINAHL, Web of Science, LILACS, SciELO, CAPES Theses and Dissertations Portal, Open Access Scientific Repository of Portugal – RCAAP, National ETD Portal, and Theses Canada), with no restrictions on language or year. Primary studies and gray literature available in full text that addressed the study objective were included; editorials, opinion pieces, and reviews were excluded. The selected studies were submitted to lexical analysis using the IRaMuTeQ software, supported by Roy’s Adaptation Theory.

**Results:**

A total of 10,875 records were identified, of which only 11 met the study objective and comprised the final corpus, totaling 641 segments analyzed. Seven classes emerged: Policy and Access; Management and Service; Community Network; Reception and Bond; Maternal-Child Care; Early Detection; and Diagnosis and Treatment. These categories highlight the complexity and interdependence among the clinical, social, and organizational dimensions of care.

**Conclusion:**

Lexical analysis revealed a representative vocabulary for nursing care of pregnant women with syphilis.

## INTRODUCTION

Syphilis during pregnancy constitutes a significant public health problem in Brazil, primarily due to its high rate of vertical transmission and severe maternal and perinatal repercussions^(1,2)^. Caring for pregnant women diagnosed with syphilis requires a comprehensive, systematic, and evidence-based approach.

In this context, nurses stand out as key professionals responsible for implementing care, surveillance, and health promotion actions^(3,4)^. Within nursing consultations and other care practices, the use of standardized and technically precise language is essential to ensure continuity and quality of care^(5)^.

However, in clinical practice, significant gaps remain in the use and documentation of terms related to the care of pregnant women with syphilis, which compromises the systematization of actions and multiprofessional communication. Moreover, concepts found in the scientific literature are not always analyzed in an integrated manner, hindering their incorporation into standardized language systems such as ICNP^®(6)^.

Lexical analysis of scientific texts enables the identification of the most recurrent concepts on the subject, revealing beliefs, practices, and dimensions of care through the lens of nursing academic discourse^(7)^. In this regard, lexicography provides a theoretical and methodological foundation for analyzing language in use, allowing not only the identification of relevant terms but also the understanding of how they reflect consolidated knowledge. By employing tools such as Descending Hierarchical Classification (DHC), it is possible to highlight semantic cores and organize them into thematic classes that represent everyday practice^(8)^.

In nursing, lexical analysis emerges as a promising strategy to understand complex phenomena such as syphilis in pregnancy. Organizing the terms that compose this field contributes to the construction of more representative terminological subsets, facilitating their incorporation into classificatory systems and promoting the standardization of language in clinical records^(9)^.

Given this, the following question arises: how are the terms related to nursing care for pregnant women with syphilis lexically organized in the scientific literature? Thus, the objective of this study was to analyze the lexicon related to nursing care for pregnant women with syphilis, based on the literature in the field.

## METHOD

### Study Design

This study is a scoping review, guided by a research protocol previously registered in the Open Science Framework (https://osf.io/cs2pe/), and conducted in accordance with the recommendations of the Joanna Briggs Institute Reviewer’s Manual and the PRISMA-ScR guidelines^(10)^. The review followed the methodological framework proposed by Arksey and O’Malley^(11)^, comprising the stages of research question identification, study selection, mapping and data extraction, and narrative synthesis of findings.

The guiding question was constructed using the PCC strategy, defined as: P (Population) – pregnant women with syphilis; C (Concept) – nursing care; C (Context) – prenatal follow-up. Accordingly, the following research question was established: What nursing care is directed toward pregnant women with syphilis?

### Study Setting

Searches were conducted in electronic databases and gray literature repositories, accessed through the CAPES Journal Portal via the Federated Academic Community (CAFe).

### Population and Selection Criteria

Publications aligned with the study objective and available in full text through the CAPES Portal were included, encompassing gray literature (theses, dissertations, and technical documents), with no restrictions on year or language. Editorials, letters to the editor, opinion articles, theoretical essays, and narrative, integrative, systematic, and scoping reviews were excluded.

### Data Collection

Study identification occurred in three phases:

- Indexed databases: PubMed/MEDLINE, CINAHL, Web of Science, LILACS, and SciELO.
- Gray literature: CAPES Theses and Dissertations Portal, Open Access Scientific Repository of Portugal (RCAAP), National ETD Portal, Theses Canada, and documents from the Brazilian Ministry of Health.
- Manual search: reference lists of included studies to identify evidence not initially retrieved.

Searches were updated in February 2025 and structured using indexed descriptors (MeSH/DeCS) and free terms, combined with Boolean operators AND and OR, adapted to each database. Main search terms included: “Syphilis,” “Syphilis, Congenital,” “Pregnant Women,” “Pregnancy,” “Prenatal Care,” “Prenatal Diagnosis,” “Nursing,” “Nursing Care,” as well as free terms such as “gestational syphilis,” “maternal syphilis,” “antenatal care,” “prenatal follow-up,” “nurse,” “nursing interventions,” “cuidado de enfermagem,” and “pré-natal.”

Study selection was performed using the Rayyan – Intelligent Systematic Review software (https://rayyan.ai/). Two researchers independently analyzed titles and abstracts; eligible studies were read in full. Disagreements were resolved by a third researcher, and duplicates were considered only once.

Data extraction was conducted by two researchers using a structured instrument in Microsoft Excel 2019^®^, with disagreements resolved by consensus. The instrument included: study identification (title, authors, year, journal, country, language), methodological aspects (design, sample size), main results, and conclusions.

### Data Analysis and Processing

Studies were classified according to the level of evidence of the Oxford Centre for Evidence-Based Medicine (OCEBM), ranging from systematic reviews of randomized clinical trials (level 1a) to expert opinion (level 5), and according to the JBI classification (levels 1 to 4). A textual corpus was constructed using excerpts from abstracts, results, and final considerations (excluding references), compiled into a single file in Microsoft Word and submitted to IRaMuTeQ.

Lexical analysis employed Descending Hierarchical Classification (DHC) and Correspondence Factor Analysis (CFA), segmenting the text into context units and grouping segments by lexical and statistical similarity. Interpretation and categorization of terms followed Roy’s Adaptation Theory, organizing data into the physiological-functional, self-concept– identity, role performance, and interdependence modes.

Results were presented through the PRISMA-ScR flowchart (identification, screening, eligibility, and inclusion), tables with methodological characteristics and main findings, charts summarizing levels of evidence and search strategies, and figures generated by IRaMuTeQ (DHC dendrogram and CFA factorial map).

### Ethical Considerations

As this study is a literature review using secondary data from public domain sources, approval by a Research Ethics Committee was not required. Copyright and reference accuracy were respected throughout the process.

## RESULTS

The review process began with the identification of 10,879 records across different databases and manual sources. Most of these records were retrieved from PubMed (n = 10,006), followed by Web of Science (n = 350), SciELO (n = 255), CAPES Journal Portal (n = 133), Cochrane (n = 61), LILACS (n = 60), and RCAAP (n = 6). In addition, four manuals/protocols from the Brazilian Ministry of Health were identified.

During the screening stage, 5,219 records were excluded for not meeting eligibility criteria, duplication, or irretrievability. Consequently, 5,656 studies were analyzed by title and abstract, resulting in the exclusion of 5,406 records due to thematic irrelevance or failure to address the guiding question.

**Figure 1:**
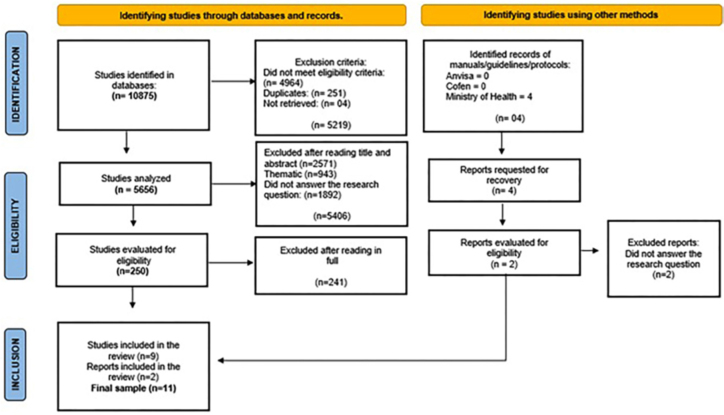
Flowchart of the study selection process. Natal, RN, Brazil, 2025.

A total of 250 studies proceeded to full-text eligibility assessment. After complete reading, 241 studies were excluded. In parallel, of the four reports/manuals evaluated through complementary methods, two were deemed ineligible. At the end of the selection process, 11 studies (nine articles and two reports/manuals) were included in the final sample of this review (Figure 1).

The final textual corpus of the study consisted of 11 documents, presented in Chart 1, which were processed and segmented into 641 text segments (TSs). All segments were considered valid for lexical analysis, resulting in full utilization (100%).

**Chart 1:** Selected studies. Natal, RN, Brazil, 2025.

Study	Country (Year)	Type of Publication/Type of Study/Level of Evidence
E1^(12)^	Brazil (2020)	Article/Qualitative, descriptive/Level of Evidence 4
E2^(13)^	Brazil (2019)	Article/Quantitative, descriptive and exploratory/Level of Evidence 4
E3^(14)^	Brazil (2019)	Article/Quantitative, descriptive and exploratory/Level of Evidence 4
E4^(15)^	Brazil (2022)	Article/Qualitative, descriptive and exploratory/Level of Evidence 4
E5^(16)^	Brazil (2021)	Article/Qualitative, descriptive/Level of Evidence 4
E6^(17)^	Mozambique (2000)	Article/Qualitative, descriptive/Level of Evidence 2c
E7^(18)^	Brazil (2020)	Article/Qualitative, action research/Level of Evidence 2c
E8^(19)^	United States (1992)	Article/Narrative descriptive review/Level of Evidence 5
E9^(20)^	United Kingdom (1998)	Article/Narrative descriptive review/Level of Evidence 5
E10^(21)^	Brazil (2007)	Technical Manual/N/A/Not specified
E11^(22)^	Brazil (2016)	Technical Manual/N/A/Not specified

After data analysis, the results were organized and presented in tables. To facilitate understanding and visualization, the studies were identified by the letter “E” (study), followed by sequential Arabic numerals (1, 2, 3, … 11), resulting in the codes E1, E2, E3, … E11.

**Figure 2:**
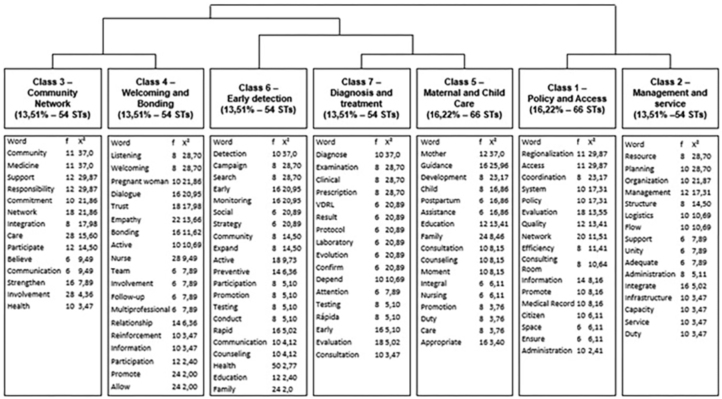
Diagram of the classes included in the dendrogram of the textual corpus from the manuscripts in the sample. Natal, RN, Brazil, 2025.

The analysis conducted using the IRaMuTeQ software allowed the classification of the segments into seven distinct thematic classes. To ensure terminological uniformity, the classes were named in the singular and standardized as follows: Class 1 – Policy and Access (16.22%); Class 2 – Management and Service (13.51%); Class 3 – Community Network (13.51%); Class 4 – Reception and Bond (13.51%); Class 5 – Maternal-Child Care (16.22%); Class 6 – Early Detection (13.51%); and Class 7 – Diagnosis and Treatment (13.51%), as shown in Figure 2.

These classes do not operate in isolation; rather, they represent the trajectory of nursing care, which begins with structural conditions (Classes 1 and 2), progresses through interpersonal relationships (Classes 3 and 4), and culminates in direct clinical intervention (Classes 6 and 7).

The relationship among the classes should be interpreted from left to right. Initially, the corpus was divided in the first iteration, separating Class 1 from the others. In the second iteration, the remaining subcorpus was subdivided into two, giving rise to Class 2 and another subcorpus. In the third iteration, this latter subcorpus was again divided, generating Class 5 on one side and a new subcorpus that, in the fourth iteration, produced Classes 3 and 4. The descending hierarchical classification was interrupted at this point, as the five classes demonstrated stability, meaning they were composed of text segments with internally homogeneous vocabulary.

**Figure 3:**
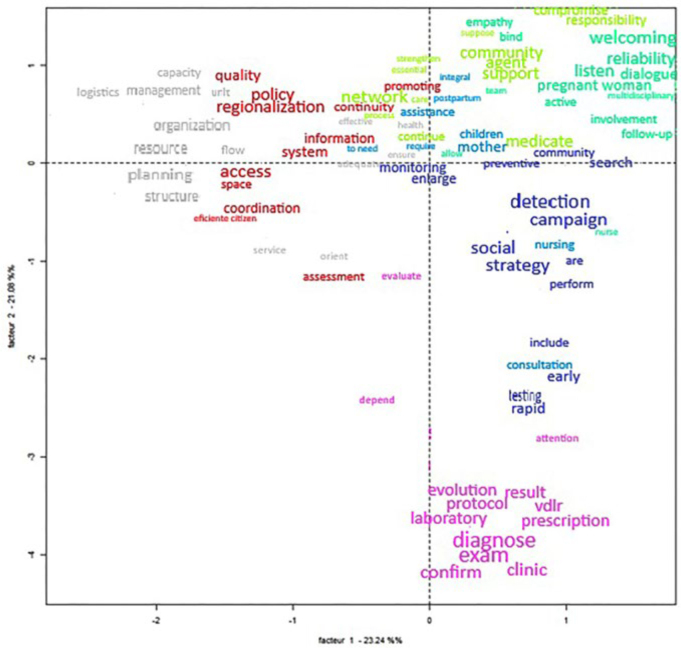
Correspondence Factor Analysis of the most frequent words in each lexical class, based on hierarchical classification. Natal, RN, Brazil, 2025.

The Correspondence Factor Analysis (CFA) mapped the spatial distribution of the classes (Figure 3). The identified classes are directly articulated with nursing clinical practice, as they translate essential dimensions of care for pregnant women with syphilis.

Lexical findings reveal that policy and management (Classes 1 and 2) impact service organization and guarantee access to prenatal care, while community network and bond (Classes 3 and 4) reinforce the importance of qualified listening and trust for adherence to treatment and partner tracing. Meanwhile, maternal-child care and early detection (Classes 5 and 6) highlight the need for integrated actions to ensure prevention of vertical transmission. In turn, diagnosis and treatment (Class 7) emphasize the technical dimension which, when dissociated from the others, may fragment care.

Thus, lexical analysis strengthens the link between scientific discourse and clinical practice, providing support for nurses to align technical interventions with the relational and organizational dimensions of care.

**Figure 4:**
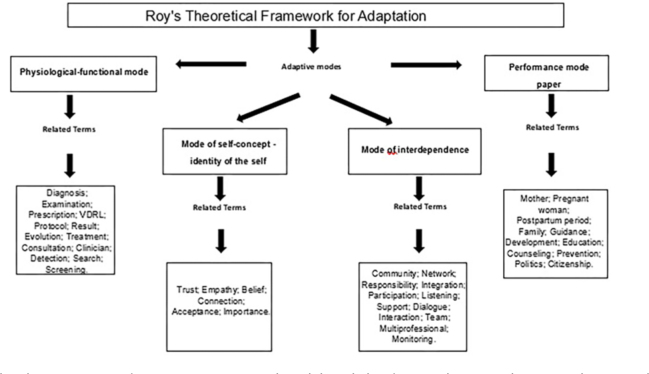
Terms related to nursing care for pregnant women with syphilis in light of Roy’s Adaptation Theory. Natal, RN, Brazil.

In Figure 4, the words extracted from the corpus were correlated with the adaptive modes of Roy’s Adaptation Theory. The visualization confirms that nursing care for pregnant women with syphilis is multidimensional: technical terms are articulated with social support terms, demonstrating that effective adaptation depends both on the nurse’s clinical competence (diagnosis and treatment) and on their ability to coordinate the care network and provide reception.

The Correspondence Factor Analysis (CFA) revealed the semantic and lexical organization of the most frequent terms in the textual corpus. As illustrated in the graph, Classes 1 (red) and 2 (gray) are grouped in the upper-left quadrant, indicating a strong correlation among the terms that compose them. This grouping, which minimally extends into the lower-left quadrant, suggests that the concepts represented by these classes are semantically central and interconnected within the study context.

In the upper-right quadrant, proximity is observed among Classes 3 (green), 4 (cyan), and 5 (blue). This spatial arrangement reflects a convergence of meanings, showing that although they are distinct classes, they share a common lexical field. Class 6 (dark blue), also located in this quadrant, subtly extends into the lower-right quadrant, suggesting a relationship—albeit more attenuated—with the predominant concepts in that region of the graph.

Finally, Class 7 (purple) stands out for its isolated position concentrated in the lower-right quadrant. This isolation indicates that the terms in this class possess semantic and lexical specificity, distinguishing them from the other groupings and representing a more particularized conceptual domain in the context of nursing care for pregnant women with syphilis.

This analysis highlights the complexity and underlying structure of the discourse on the subject, revealing patterns of association among terms and concepts in a clear and visual manner.

## DISCUSSION

Lexical analysis highlighted the discursive construction of the phenomenon of syphilis in pregnancy. From the grouping of words, semantic cores were identified that express different conceptual axes of care. Although the organization resulted from systematic software processing, the clinical inference was grounded in the researchers’ interpretation^(8)^.

The seven classes were standardized according to the semantic axes they represent: Policy and Access, Management and Service, Community Network, Reception and Bond, Maternal-Child Care, Early Detection, and Diagnosis and Treatment. Each class brings together interrelated terms that, collectively, deepen the understanding of barriers and strategies in preventing vertical transmission.

The analysis was based on Callista Roy’s Adaptation Theory, which views the individual as an adaptive system responding to internal and external stimuli^(23)^. From this perspective, the terms of the classes manifest adaptive processes and challenges imposed by syphilis, reflecting both care demands and coping strategies^(24)^. It is the nurse’s responsibility to recognize these needs in order to promote adaptive balance, aiming for safe pregnancy and the prevention of adverse outcomes for the mother–conceptus dyad^(3)^.

CFA demonstrated proximity between Class 1 – Policy and Access (terms: “regionalization,” “coordination,” “access”) and Class 2 – Management and Service (terms: “resources,” “planning,” “organization”). This connection indicates that public policy formulation is inseparable from the organizational capacity of services. Recent data show that the prevalence of syphilis in pregnancy reached 1.02% in 2019, with a significant increase in previous years. This scenario reflects structural weaknesses in the healthcare network. The literature confirms that failures in planning and resource allocation hinder policy implementation, limit treatment adherence, and compromise comprehensive care^(4)^. Thus, the conceptual overlap between the classes reinforces the need for more efficient and articulated policies capable of addressing inequalities and ensuring maternal and child health^(1,2)^.

The analysis also revealed a strong connection between Class 3 – Community Network and Class 4 -Reception and Bond. The proximity of terms such as “community” and “network” with “listening” and “trust” demonstrates that care for pregnant women with syphilis goes beyond the clinical dimension. Prenatal care in Primary Health Care is a privileged moment for case identification, notification, and treatment^(25)^. In this context, reception and qualified listening become central tools for building trust and bonds, favoring adherence of pregnant women and their partners to treatment^(6)^. The literature also emphasizes the relevance of Community Health Agents (CHAs) in the Family Health Strategy (FHS), whose role contributes to the early identification of pregnant women and health education within the community^(26)^.

Therefore, addressing gestational syphilis must integrate relational, community, and social dimensions. This integration promotes trust, strengthens bonds, and reduces barriers to accessing health services^(1,2)^.

CFA revealed overlap between Class 5 – Maternal-Child Care and Class 6 – Early Detection. In Class 5, terms such as “mother” and “child” emphasize the focus on the dyad and continuity of care. The word “development” points to the relevance of the affective bond, which may be impacted by diagnosis, while “guidance” plays a central role in empowering pregnant women. In parallel, Class 6 gathers terms such as “campaign” and “search,” which reflect the public health pillar of proactive action. “Active search” and “early detection” reinforce the need for agile policies capable of overcoming passive waiting for diagnosis. The overlap of these classes demonstrates that maternal-child care and early detection are interconnected dimensions, forming a unified strategy that depends on bonding, reception, and proactive health services^(27)^.

Class 7 – Diagnosis and Treatment appears in an isolated and concentrated position, marked by technical terms such as “examination” and “prescription.” This separation is not merely graphical but reflects a conceptual dissociation between clinical procedures and the social and human context of pregnant women.

In summary, the results suggest that success in addressing gestational syphilis goes beyond the mere implementation of protocols. It requires articulation between detection and treatment actions (biological), humanized care, practices of reception and bonding (psychosocial), as well as integrated networks, organized and accessible services (management). This integration aligns technical interventions with the social and human dimensions of care, providing support for more precise nursing diagnoses and effective interventions in prenatal care.

## CONCLUSION

The lexical analysis of terms related to nursing care for pregnant women with syphilis, in light of the nursing literature, allowed the identification of the most recurrent vocabulary concerning the phenomenon. With the support of the IRaMuTeQ software, a set of terms strongly associated with the theme was highlighted.

The most relevant words were: policy, access, management, services, network, community, reception, bond, care, mother, child, detection, campaign, search, diagnosis, examination, and prescription.

This study of the lexicon of gestational syphilis may contribute to the improvement of nurses’ clinical and critical reasoning. By providing scientific support, it enables the identification of challenges and care strategies, assisting in the formulation of more accurate nursing diagnoses and in the implementation of interventions that consider the clinical, social, and human dimensions of pregnant women.

It is recommended that future studies deepen the analysis of the interrelationship among these terms in order to validate and expand the results obtained.

## DATA AVAILABILITY

The entire dataset supporting the results of this study is available upon request to the corresponding author.
