# Supplementary material for: Lexical analysis of terms related to nursing care for pregnant women with syphilis
Source: Rev Esc Enferm USP. 2026 May 1;60:e20250386. doi: 10.1590/1980-220X-REEUSP-2025-0386en (PMC13138794; doi:10.1590/1980-220X-REEUSP-2025-0386en)
Supplement: Translation

# Translation: Análise léxica dos termos relacionados ao cuidado de enfermagem à gestante com sífilis

**DOI:** 10.1590/1980-220X-REEUSP-2025-0386pt

**Authors:** Ana Beatriz Pereira da Silva, Richardson Augusto Rosendo da Silva, Jocellem Alves de Medeiros, José Leonildo Fernandes de Queiroz, Vitória de Araújo e Silva, Amanda de Brito Rangel Pereira Sales, Jéssica Fernanda Galon, Elise Cristina dos Santos Felix

**Affiliations:** 1 Universidade Federal do Rio Grande do Norte, Programa de Pós-Graduação em Enfermagem, Natal, RN, Brasil.; 2 Universidade Federal do Rio Grande do Norte, Departamento de Enfermagem, Natal, RN, Brasil.; 3 Universidade de Ribeirão Preto, Departamento de Enfermagem, Ribeirão Preto, SP, Brasil.

**Keywords:** Sífilis, Gestantes, Cuidados de Enfermagem, Cuidado Pré-Natal, Terminologia Padronizada em Enfermagem

## Abstract

**Objetivo:**

Analisar o léxico relacionado ao cuidado de enfermagem à gestante com sífilis, a partir da literatura científica.

**Método:**

Scoping review conduzida conforme o referencial de Arksey e O’Malley e as diretrizes PRISMA-ScR. As buscas foram realizadas em bases nacionais e internacionais (PubMed, CINAHL, Web of Science, LILACS, SciELO, Portal de Teses e Dissertações da CAPES, Repositório Científico de Acesso Aberto de Portugal – RCAAP, National ETD Portal e Theses Canada), sem restrição de idioma ou ano. Foram incluídos estudos primários e literatura cinzenta disponíveis na íntegra que respondessem ao objetivo; excluíram-se editoriais, opiniões e revisões. Os estudos selecionados foram submetidos à análise lexical pelo software IRaMuTeQ, com suporte da Teoria da Adaptação de Roy.

**Resultados:**

Identificaram-se 10.875 registros, dos quais apenas 11 atenderam ao objetivo do estudo e compuseram o corpus final, totalizando 641 segmentos analisados. Emergiram sete classes: Política e Acesso; Gestão e Serviço; Rede Comunitária; Acolhimento e Vínculo; Cuidado Materno-Infantil; Detecção Precoce; e Diagnóstico e Tratamento. Essas categorias evidenciam a complexidade e a interdependência entre as dimensões clínicas, sociais e organizacionais do cuidado.

**Conclusão:**

A análise lexical revelou um vocabulário representativo para o cuidado à gestante com sífilis.

## INTRODUÇÃO

A sífilis na gestação constitui um relevante problema de saúde pública no Brasil, sobretudo em razão da elevada taxa de transmissão vertical e das graves repercussões maternas e perinatais^(1,2)^. O cuidado à gestante com diagnóstico de sífilis requer uma abordagem integral, sistematizada e fundamentada em evidências.

Nesse contexto, o enfermeiro destaca-se como um dos principais profissionais responsáveis pela execução das ações de cuidado, vigilância e promoção da saúde^(3,4)^. No âmbito da consulta de enfermagem e das demais práticas assistenciais, a utilização de uma linguagem padronizada e tecnicamente precisa é essencial para assegurar a continuidade e a qualidade do atendimento^(5)^.

Entretanto, observa-se que, na prática clínica, persistem lacunas significativas no uso e registro dos termos relacionados ao cuidado às gestantes com sífilis, o que compromete a sistematização das ações e a comunicação multiprofissional. Ademais, os conceitos presentes na literatura científica nem sempre são analisados de forma integrada, dificultando sua incorporação em sistemas de linguagem padronizada, como a CIPE^®(6)^.

A análise lexical de textos científicos possibilita identificar os conceitos mais recorrentes sobre o tema, revelando crenças, práticas e dimensões do cuidado à luz dos discursos acadêmicos da enfermagem^(7)^. Nesse sentido, a lexicografia oferece base teórica e metodológica para a análise da linguagem em uso, permitindo não apenas a identificação de termos relevantes, mas também a compreensão de como refletem saberes consolidados. Com o emprego de ferramentas como a Classificação Hierárquica Descendente (CHD), torna-se possível evidenciar núcleos de sentido e organizá-los em classes temáticas que representam o cotidiano da prática^(8)^.

Na enfermagem, a análise lexical configura-se como estratégia promissora para compreender fenômenos complexos, como a sífilis na gestação. A organização dos termos que compõem esse campo contribui para a construção de subconjuntos terminológicos mais representativos, favorecendo sua incorporação em sistemas classificatórios e a padronização da linguagem nos registros clínicos^(9)^.

Diante disso, questiona-se: como os termos relacionados ao cuidado de enfermagem à gestante com sífilis estão organizados lexicalmente na literatura científica? Assim, o objetivo deste estudo foi analisar o léxico relacionado ao cuidado de enfermagem à gestante com sífilis, a partir da literatura da área.

## MÉTODO

### Desenho do Estudo

Trata-se de uma *scoping review*, orientada por protocolo de pesquisa previamente registrado no *Open Science Framework* (https://osf.io/cs2pe/), conduzida conforme as recomendações do *Joanna Briggs Institute Reviewer’s Manual* e as diretrizes PRISMA-ScR^(10)^. A revisão seguiu o referencial metodológico de Arksey e O’Malley^(11)^, composto pelas etapas de identificação da questão de pesquisa, seleção dos estudos, mapeamento e extração dos resultados, além da síntese narrativa dos achados.

A questão norteadora foi construída pela estratégia PCC, definida como: P (*Population*) – mulheres gestantes com sífilis; C (*Concept*) – cuidados de enfermagem; C (*Context*) – acompanhamento gestacional. Assim, estabeleceu-se a seguinte pergunta: quais os cuidados de enfermagem direcionados às gestantes com sífilis?

### Local do Estudo

As buscas foram realizadas em bases de dados eletrônicas e repositórios de literatura cinzenta, por meio do Portal de Periódicos da CAPES, acessado via Comunidade Acadêmica Federada (CAFe).

### População e Critérios de Seleção

Foram incluídas publicações alinhadas ao objetivo da pesquisa e disponíveis na íntegra pelo Portal CAPES, incluindo literatura cinzenta (teses, dissertações e documentos técnicos), sem restrição de ano ou idioma. Excluíram-se editoriais, cartas ao editor, artigos de opinião, ensaios teóricos e revisões narrativas, integrativas, sistemáticas e de escopo.

### Coleta de Dados

A identificação de estudos ocorreu em três fases:

- Bases indexadas: PubMed/MEDLINE, CINAHL, Web of Science, LILACS e SciELO.
- Literatura cinzenta: Portal de Teses e Dissertações da CAPES, Repositório Científico de Acesso Aberto de Portugal (RCAAP), National ETD Portal, Theses Canada e documentos do Ministério da Saúde do Brasil.
- Busca manual: listas de referências dos estudos incluídos, para identificar evidências não recuperadas inicialmente.

As buscas foram atualizadas em fevereiro de 2025 e estruturadas com descritores indexados (*MeSH*/DeCS) e termos livres, combinados com os operadores booleanos *AND* e *OR*, adaptados a cada base. Principais chaves de busca: “Syphilis”, “Syphilis, Congenital”, “Pregnant Women”, “Pregnancy”, “Prenatal Care”, “Prenatal Diagnosis”, “Nursing”, “Nursing Care”, além de termos livres como “gestational syphilis”, “maternal syphilis”, “antenatal care”, “prenatal follow-up”, “nurse”, “nursing interventions”, “cuidado de enfermagem” e “pré-natal”.

A seleção foi realizada software *Rayyan – Intelligent Systematic Review* (https://rayyan.ai/). Dois pesquisadores, de forma independente, analisaram títulos e resumos; os estudos elegíveis foram lidos na íntegra. Divergências foram resolvidas por um terceiro pesquisador, e duplicatas foram consideradas apenas uma vez.

A extração dos dados foi conduzida pelos dois pesquisadores por meio de instrumento estruturado no *Microsoft Excel* 2019^®^, com resolução de divergências por consenso. O instrumento contemplou: identificação do estudo (título, autores, ano, periódico, país, idioma), aspectos metodológicos (delineamento, tamanho da amostra), principais resultados e conclusões.

### Análise e Tratamento dos Dados

Os estudos foram classificados segundo o nível de evidência do *Oxford Centre for Evidence-Based Medicine* (OCEBM), variando de revisões sistemáticas de ensaios clínicos randomizados (nível 1a) a opinião de especialistas (nível 5), e segundo a classificação do JBI (níveis 1 a 4). Constituiu-se um corpus textual com trechos de resumos, resultados e considerações finais (excluídas referências), reunido em arquivo único no *Microsoft Word* e submetido ao IRaMuTeQ.

A análise lexical empregou a Classificação Hierárquica Descendente (CHD) e a Análise Fatorial de Correspondência (AFC), segmentando o texto em unidades de contexto e agrupando segmentos por similaridade lexical e estatística. A interpretação e categorização dos termos seguiu a Teoria da Adaptação de Roy, organizando os dados nos modos fisiológico-funcional, autoconceito-identidade, desempenho de papel e interdependência.

Os resultados foram apresentados por meio do fluxograma *PRISMA-ScR* (identificação, triagem, elegibilidade e inclusão), tabelas com características metodológicas e principais achados, quadros com síntese do nível de evidência e estratégias de busca, além de figuras geradas pelo IRaMuTeQ (dendro-grama da CHD e mapa fatorial da AFC).

### Aspectos Éticos

Por tratar-se de uma revisão de literatura com dados secundários de domínio público, o estudo dispensa aprovação por Comitê de Ética em Pesquisa, respeitando os direitos autorais e a fidedignidade das referências utilizadas.

## RESULTADOS

O processo de revisão iniciou-se com a identificação de 10.879 registros em diferentes bases de dados e fontes manuais. A maior parte desses registros foi obtida no PubMed (n = 10.006), seguida pelo Web of Science (n = 350), SciELO (n = 255), Portal de Periódicos CAPES (n = 133), Cochrane (n = 61), LILACS (n = 60) e RCAAP (n = 6). Adicionalmente, foram identificados quatro manuais/protocolos provenientes do Ministério da Saúde.

Na etapa de triagem, 5.219 registros foram excluídos por não atenderem aos critérios de elegibilidade, duplicidade ou por serem irrecuperáveis. Dessa forma, 5.656 estudos foram analisados quanto ao título e resumo, resultando na exclusão de 5.406 registros por irrelevância temática ou por não responderem à questão norteadora.

Um total de 250 estudos seguiu para a avaliação completa da elegibilidade. Após a leitura do texto na íntegra, 241 estudos foram excluídos. Paralelamente, dos quatro relatórios/manuais avaliados por métodos complementares, dois foram considerados inelegíveis. Ao final do processo de seleção, 11 estudos (nove artigos e dois relatórios/manuais) foram incluídos na amostra final desta revisão (Figura 1).

**Figura 1:** Esquema do processo de seleção dos estudos. Natal, RN, Brasil, 2025.

O corpus textual final da pesquisa foi constituído por 11 documentos, apresentados no Quadro 1, os quais foram processados e segmentados em 641 segmentos de texto (STs). Todos os segmentos foram considerados válidos para a análise lexical, resultando em aproveitamento total (100%).

**Quadro 1:** Estudos selecionados – Natal, RN, Brasil, 2025

Estudo	País (Ano)	Tipo de publicação/Tipo de estudo/Nível de Evidência
E1^(12)^	Brasil (2020)	Artigo/Qualitativo, descritivo/Nível de Evidência 4
E2^(13)^	Brasil (2019)	Artigo/Quantitativo, descritivo e exploratório/Nível de Evidência 4
E3^(14)^	Brasil (2019)	Artigo/Quantitativo, descritivo e exploratório/Nível de Evidência 4
E4^(15)^	Brasil (2022)	Artigo/Qualitativo, descritivo e exploratório/Nível de Evidência 4
E5^(16)^	Brasil (2021)	Artigo/Qualitativo, descritivo/Nível de Evidência 4
E6^(17)^	Mozambique (2000)	Artigo/Qualitativo, descritivo/Nível de Evidência 2c
E7^(18)^	Brasil (2020)	Artigo/Qualitativo, pesquisa-ação/Nível de Evidência 2c
E8^(19)^	Estados Unidos (1992)	Artigo/Revisão narrativa descritiva/Nível de Evidência 5
E9^(20)^	Reino Unido (1998)	Artigo/Revisão narrativa descritiva/Nível de Evidência 5
E10^(21)^	Brasil (2007)	Manual Técnico/N/A/Não especificado
E11^(22)^	Brasil (2016)	Manual Técnico/N/A/Não especificado

Após a análise dos dados, estes foram organizados e apresentados em tabelas. Para facilitar a compreensão e a visualização, os estudos foram identificados pela letra “E” (estudo), seguida por números arábicos sequenciais (1, 2, 3, … 11), originando as codificações E1, E2, E3, … E11.

A análise conduzida pelo software IRaMuTeQ permitiu a classificação dos segmentos em sete classes temáticas distintas. Para garantir uniformidade terminológica, as classes foram nomeadas no singular e padronizadas da seguinte forma: Classe 1 – Política e Acesso (16,22%); Classe 2 – Gestão e Serviço (13,51%); Classe 3 – Rede Comunitária (13,51%); Classe 4 – Acolhimento e Vínculo (13,51%); Classe 5 – Cuidado Materno-Infantil (16,22%); Classe 6 – Detecção Precoce (13,51%); e Classe 7 – Diagnóstico e Tratamento (13,51%), conforme descrito na Figura 2.

**Figura 2:** Diagrama das classes integrantes do dendograma do corpus textual referente aos manuscritos da amostra. Natal, RN, Brasil, 2025.

Essas classes não operam de maneira isolada; ao contrário, representam a trajetória do cuidado de enfermagem, que se inicia nas condições estruturais (Classes 1 e 2), perpassa a relação interpessoal (Classes 3 e 4) e culmina na intervenção clínica direta (Classes 6 e 7).

A relação entre as classes deve ser interpretada da esquerda para a direita. Inicialmente, o corpus foi dividido na primeira iteração, separando a Classe 1 das demais. Na segunda iteração, o subcorpus remanescente foi subdividido em dois, originando a Classe 2 e outro subcorpus. Na terceira iteração, esse último subcorpus foi novamente dividido, gerando a Classe 5 de um lado e um novo subcorpus que, na quarta iteração, deu origem às Classes 3 e 4. A classificação hierárquica descendente foi interrompida nesse ponto, uma vez que as cinco classes apresentaram estabilidade, ou seja, foram compostas por segmentos de texto com vocabulário internamente homogêneo.

A Análise Fatorial de Correspondência (AFC) mapeou a distribuição espacial das classes (Figura 3). As classes identificadas articulam-se diretamente com a prática clínica da enfermagem, ao traduzirem dimensões essenciais do cuidado à gestante com sífilis.

**Figura 3:** Análise Fatorial de Correspondência das palavras mais frequentes em cada classe lexical, com base em uma classificação hierárquica. Natal, RN, Brasil, 2025.

Os achados lexicais revelam que políticas e gestão (Classes 1 e 2) impactam a organização dos serviços e a garantia de acesso ao pré-natal, enquanto rede comunitária e vínculo (Classes 3 e 4) reforçam a importância da escuta qualificada e da confiança para adesão ao tratamento e rastreamento de parceiros. Já o cuidado materno-infantil e a detecção precoce (Classes 5 e 6) evidenciam a necessidade de ações integradas que assegurem a prevenção da transmissão vertical. Por sua vez, diagnóstico e tratamento (Classe 7) destacam a dimensão técnica que, quando dissociada das demais, pode fragmentar a assistência.

Dessa forma, a análise lexical fortalece o vínculo entre discurso científico e prática clínica, oferecendo subsídios para que o enfermeiro alinhe intervenções técnicas às dimensões relacionais e organizacionais do cuidado.

Na Figura 4, as palavras extraídas do corpus foram correlacionadas aos modos adaptativos da Teoria de Roy. A visualização confirma que o cuidado à gestante com sífilis é multidimensional: termos técnicos articulam-se com termos de suporte social, evidenciando que a adaptação eficaz depende tanto da competência clínica do enfermeiro (diagnóstico e tratamento) quanto da sua capacidade de articulação da rede e de acolhimento.

**Figura 4:** Termos relacionados ao cuidado à gestante com sífilis à luz do referencial teórico de adaptação de Roy. Natal, RN, Brasil.

A Análise Fatorial de Correspondência (AFC) revelou a organização semântica e lexical dos termos mais frequentes no corpus textual. Conforme ilustrado no gráfico, as Classes 1 (vermelha) e 2 (cinza) encontram-se agrupadas no quadrante superior esquerdo, indicando forte correlação entre os termos que as compõem. Esse agrupamento, que se estende minimamente para o quadrante inferior esquerdo, sugere que os conceitos representados por essas classes são semanticamente centrais e interligados no contexto do estudo.

No quadrante superior direito, observa-se a proximidade entre as Classes 3 (verde), 4 (ciano) e 5 (azul). Tal disposição espacial reflete uma convergência de significados, evidenciando que, embora sejam classes distintas, compartilham um campo lexical comum. A Classe 6 (azul-escuro), também localizada nesse quadrante, avança sutilmente para o quadrante inferior direito, sugerindo uma relação, ainda que mais atenuada, com os conceitos predominantes nessa região do gráfico.

Por fim, a Classe 7 (roxa) destaca-se por sua posição isolada e concentrada no quadrante inferior direito. Esse isolamento indica que os termos dessa classe possuem especificidade semântica e lexical, diferenciando-se dos demais agrupamentos e representando um domínio conceitual mais particularizado no âmbito do cuidado à gestante com sífilis.

Essa análise evidencia a complexidade e a estrutura subjacente do discurso sobre o tema, revelando padrões de associação entre termos e conceitos de forma clara e visual.

## DISCUSSÃO

A análise lexical evidenciou a construção discursiva do fenômeno da sífilis na gestação. A partir do agrupamento dos vocábulos, identificaram-se núcleos de sentido que expressam diferentes eixos conceituais do cuidado. Embora a organização decorra do processamento sistemático do software, a inferência clínica fundamentou-se na interpretação dos pesquisadores^(8)^.

As sete classes foram padronizadas conforme os eixos semânticos que representam: Política e Acesso, Gestão e Serviço, Rede Comunitária, Acolhimento e Vínculo, Cuidado Materno-Infantil, Detecção Precoce e Diagnóstico e Tratamento. Cada classe reúne termos inter-relacionados que, em conjunto, aprofundam a compreensão sobre barreiras e estratégias na prevenção da transmissão vertical.

A análise foi fundamentada na Teoria da Adaptação de Callista Roy, que compreende o indivíduo como um sistema adaptativo que responde a estímulos internos e externos^(23)^. Nessa perspectiva, os termos das classes manifestam processos adaptativos e desafios impostos pela sífilis, refletindo tanto demandas de cuidado quanto estratégias de enfrenta-mento^(24)^. Cabe ao enfermeiro reconhecer essas necessidades para promover o equilíbrio adaptativo, visando uma gestação segura e a prevenção de desfechos adversos para a díade mãe-concepto^(3)^.

A AFC demonstrou proximidade entre a Classe 1 – Política e Acesso (termos: “regionalização”, “coordenação”, “acesso”) e a Classe 2 – Gestão e Serviço (termos: “recursos”, “planejamento”, “organização”). Tal conexão indica que a formulação de políticas públicas é indissociável da capacidade organizacional dos serviços. Dados recentes apontam que a prevalência da sífilis na gestação atingiu 1,02% em 2019, com aumento expressivo nos anos anteriores. Esse cenário reflete fragilidades estruturais na rede de atenção. A literatura confirma que falhas no planejamento e na alocação de recursos dificultam a implementação de políticas, limitam a adesão ao tratamento e comprometem a integralidade do cuidado^(4)^. Assim, a sobreposição conceitual entre as classes reforça a necessidade de políticas mais eficientes e articuladas, capazes de enfrentar desigualdades e garantir a saúde materno-infantil^(1,2)^.

A análise também revelou forte conexão entre a Classe 3 – Rede Comunitária e a Classe 4 – Acolhimento e Vínculo. A proximidade de termos como “comunitário” e “rede” com “escutar” e “confiança” demonstra que o cuidado à gestante com sífilis ultrapassa a dimensão clínica. O pré-natal na Atenção Básica é um momento privilegiado para identificação, notificação e tratamento dos casos^(25)^. Nesse contexto, o acolhimento e a escuta qualificada tornam-se ferramentas centrais para a construção do vínculo e da confiança, favorecendo a adesão das gestantes e de seus parceiros ao tratamento^(6)^. A literatura também ressalta a relevância dos Agentes Comunitários de Saúde (ACS) na Estratégia Saúde da Família (ESF), cuja atuação contribui para a captação precoce de gestantes e para a educação em saúde no âmbito comunitário^(26)^.

Portanto, o enfrentamento da sífilis gestacional deve integrar dimensões relacionais, comunitárias e sociais. Essa integração promove confiança, fortalece vínculos e reduz barreiras de acesso aos serviços de saúde^(1,2)^.

A AFC evidenciou sobreposição da Classe 5 – Cuidado Materno-Infantil com a Classe 6 – Detecção Precoce. Na Classe 5, termos como “mãe” e “criança” ressaltam o foco na díade e a continuidade do cuidado. O vocábulo “desenvolvimento” aponta para a relevância do vínculo afetivo, que pode ser impactado pelo diagnóstico, enquanto “orientação” assume papel central no empoderamento da gestante. Paralelamente, a Classe 6 reúne termos como “campanha” e “busca”, que refletem o pilar da saúde pública: a ação proativa. A “busca ativa” e a “detecção precoce” reforçam a necessidade de políticas ágeis, capazes de superar a espera passiva pelo diagnóstico. A sobreposição dessas classes demonstra que o cuidado materno-infantil e a detecção precoce são dimensões interligadas, formando uma estratégia única que depende do vínculo, do acolhimento e da proatividade dos serviços de saúde^(27)^.

A Classe 7 – Diagnóstico e Tratamento aparece em posição isolada e concentrada, marcada por termos técnicos como “exame” e “prescrição”. Essa separação não é apenas gráfica, mas reflete uma dissociação conceitual entre procedimentos clínicos e o contexto social e humano da gestante.

Em síntese, os resultados sugerem que o sucesso no enfrentamento da sífilis gestacional ultrapassa a mera implementação de protocolos. É necessária a articulação entre ações de detecção e tratamento (biológicas), cuidado humanizado, práticas de acolhimento e vínculo (psicossociais), além de rede integrada, serviços organizados e acessíveis (gestão). Essa integração alinha intervenções técnicas às dimensões sociais e humanas do cuidado, oferecendo subsídios para diagnósticos de enfermagem mais precisos e intervenções efetivas na atenção pré-natal.

## CONCLUSÃO

A análise lexical dos termos relacionados ao cuidado da gestante com sífilis, à luz da literatura de enfermagem, permitiu identificar o vocabulário mais recorrente sobre o fenômeno. Com o auxílio do software IRaMuTeQ, evidenciou-se um conjunto de termos fortemente associados ao tema.

Os vocábulos mais relevantes foram: políticas, acesso, gestão, serviços, rede, comunitário, acolhimento, vínculo, cuidado, mãe, criança, detecção, campanha, busca, diagnóstico, exame e prescrição.

Este estudo sobre o léxico da sífilis gestacional pode contribuir para o aprimoramento do raciocínio clínico e crítico do enfermeiro. Ao fornecer respaldo científico, possibilita identificar desafios e estratégias de cuidado, auxiliando na formulação de diagnósticos de enfermagem mais precisos e na aplicação de intervenções que considerem as dimensões clínica, social e humana da gestante.

Recomenda-se que pesquisas futuras aprofundem a análise da inter-relação entre esses termos, a fim de validar e expandir os resultados obtidos.

## DISPONIBILIDADE DE DADOS

Todo o conjunto de dados que dá suporte aos resultados deste estudo está disponível mediante solicitação ao autor correspondente.

# Translation: Análisis léxico de los términos relacionados con el cuidado de enfermería a la gestante con sífilis

**DOI:** 10.1590/1980-220X-REEUSP-2025-0386es

**Authors:** Ana Beatriz Pereira da Silva, Richardson Augusto Rosendo da Silva, Jocellem Alves de Medeiros, José Leonildo Fernandes de Queiroz, Vitória de Araújo e Silva, Amanda de Brito Rangel Pereira Sales, Jéssica Fernanda Galon, Elise Cristina dos Santos Felix

**Affiliations:** 1 Universidade Federal do Rio Grande do Norte, Programa de Pós-Graduação em Enfermagem, Natal, RN, Brasil.; 2 Universidade Federal do Rio Grande do Norte, Departamento de Enfermagem, Natal, RN, Brasil.; 3 Universidade de Ribeirão Preto, Departamento de Enfermagem, Ribeirão Preto, SP, Brasil.

**Keywords:** Sífilis, Personas Embarazadas, Atención de Enfermería, Atención Prenatal, Terminología Normalizada de Enfermería

## Abstract

**Objetivo:**

Analizar el léxico relacionado con el cuidado de enfermería a la gestante con sífilis, a partir de la literatura científica.

**Método:**

Scoping review realizada conforme al marco de referencia de Arksey y O’Malley y las directrices PRISMA-ScR. Las búsquedas se efectuaron en bases nacionales e internacionales (PubMed, CINAHL, Web of Science, LILACS, SciELO, Portal de Tesis y Disertaciones de CAPES, Repositorio Científico de Acceso Abierto de Portugal – RCAAP, National ETD Portal y Theses Canada), sin restricción de idioma ni de año. Se incluyeron estudios primarios y literatura gris disponibles en su totalidad que respondieran al objetivo; se excluyeron editoriales, opiniones y revisiones. Los estudios seleccionados fueron sometidos al análisis léxico mediante el software IRaMuTeQ, con apoyo de la Teoría de la Adaptación de Roy.

**Resultados:**

Se identificaron 10.875 registros, de los cuales solo 11 cumplieron el objetivo del estudio y conformaron el corpus final, totalizando 641 segmentos analizados. Emergieron siete clases: Política y Acceso; Gestión y Servicio; Red Comunitaria; Acogida y Vínculo; Cuidado Materno-Infantil; Detección Precoz; y Diagnóstico y Tratamiento. Estas categorías evidencian la complejidad y la interdependencia entre las dimensiones clínicas, sociales y organizacionales del cuidado.

**Conclusión:**

El análisis léxico reveló un vocabulario representativo para el cuidado de la gestante con sífilis.

## INTRODUCCIÓN

La sífilis durante el embarazo constituye un problema de salud pública relevante en Brasil, sobre todo debido a la elevada tasa de transmisión vertical y a las graves repercusiones maternas y perinatales^(1,2)^. La atención a la gestante con diagnóstico de sífilis requiere un enfoque integral, sistematizado y basado en la evidencia.

En este contexto, el enfermero se destaca como uno de los principales profesionales responsables de la ejecución de las acciones de cuidado, vigilancia y promoción de la salud^(3,4)^. En el ámbito de la consulta de enfermería y de las demás prácticas asistenciales, el uso de un lenguaje estandarizado y técnicamente preciso es esencial para asegurar la continuidad y la calidad de la atención^(5)^.

Sin embargo, se observa que, en la práctica clínica, persisten lagunas significativas en el uso y registro de los términos relacionados con la atención a las gestantes con sífilis, lo que compromete la sistematización de las acciones y la comunicación multiprofesional. Además, los conceptos presentes en la literatura científica no siempre se analizan de manera integrada, lo que dificulta su incorporación en sistemas de lenguaje estandarizado, como el CIPE^®(6)^.

El análisis léxico de textos científicos permite identificar los conceptos más recurrentes sobre el tema, revelando creencias, prácticas y dimensiones de la atención a la luz de los discursos académicos de enfermería^(7)^. En este sentido, la lexicografía ofrece una base teórica y metodológica para el análisis del lenguaje en uso, permitiendo no solo la identificación de términos relevantes, sino también la comprensión de cómo reflejan conocimientos consolidados. Con el empleo de herramientas como la CJD, es posible evidenciar núcleos de sentido y organizarlos en clases temáticas que representan el día a día de la práctica^(8)^.

En enfermería, el análisis léxico se configura como una estrategia prometedora para comprender fenómenos complejos, como la sífilis en el embarazo. La organización de los términos que componen este campo contribuye a la construcción de subconjuntos terminológicos más representativos, favoreciendo su incorporación en sistemas clasificatorios y la estandarización del lenguaje en los registros clínicos^(9)^.

Ante esto, se plantea la pregunta: ¿cómo se organizan léxicamente en la literatura científica los términos relacionados con la atención de enfermería a la gestante con sífilis? Así, el objetivo de este estudio fue analizar el léxico relacionado con la atención de enfermería a la gestante con sífilis, a partir de la literatura del área.

## MÉTODO

### Diseño del Estudio

Se trata de una revisión de alcance (*scoping review)*, guiada por un protocolo de investigación previamente registrado en el *Open Science Framework* (https://osf.io/cs2pe/), realizada de acuerdo con las recomendaciones del *Joanna Briggs Institute Reviewer’s Manual* y las directrices PRISMA-ScR^(10)^. La revisión siguió el marco metodológico de Arksey y O’Malley^(11)^, compuesto por las etapas de identificación de la pregunta de investigación, selección de los estudios, mapeo y extracción de los resultados, además de la síntesis narrativa de los hallazgos.

La pregunta orientadora se construyó mediante la estrategia PCC, definida como: P (*Población*) – mujeres embarazadas con sífilis; C (*Concepto*) – cuidados de enfermería; C (*Contexto*) – seguimiento del embarazo. Así, se estableció la siguiente pregunta: ¿cuáles son los cuidados de enfermería dirigidos a las mujeres embarazadas con sífilis?

### Lugar del Estudio

Las búsquedas se realizaron en bases de datos electrónicas y repositorios de literatura gris, a través del Portal de Periódicos de CAPES, al que se accedió vía la Comunidad Académica Federada (CAFe).

### Población y Criterios de Selección

Se incluyeron publicaciones alineadas con el objetivo de la investigación y disponibles en su totalidad a través del Portal CAPES, incluyendo literatura gris (tesis, disertaciones y documentos técnicos), sin restricción de año o idioma. Se excluyeron editoriales, cartas al editor, artículos de opinión, ensayos teóricos y revisiones narrativas, integrativas, sistemáticas y de alcance.

### Recolección de Datos

La identificación de los estudios se llevó a cabo en tres fases:

- Bases de datos indexadas: PubMed/MEDLINE, CINAHL, Web of Science, LILACS y SciELO.
- Literatura gris: Portal de Tesis y Disertaciones de CAPES, Repositorio Científico de Acceso Abierto de Portugal (RCAAP), National ETD Portal, Theses Canada y documentos del Ministerio de Salud de Brasil.
- Búsqueda manual: listas de referencias de los estudios incluidos, para identificar la evidencia no recuperada inicialmente.

Las búsquedas se actualizaron en febrero de 2025 y se estructuraron con descriptores indexados (*MeSH*/DeCS) y términos libres, combinados con los operadores booleanos *AND* y *OR*, adaptados a cada base. Principales palabras clave de búsqueda: “Syphilis”, “Syphilis, Congenital”, “Pregnant Women”, “Pregnancy”, “Prenatal Care”, “Prenatal Diagnosis”, “Nursing”, “Nursing Care”, además de términos libres como “gestational syphilis”, “maternal syphilis”, “antenatal care”, “prenatal followup”, “nurse”, “nursing interventions”, “cuidado de enfermagem” y “pré-natal”.

La selección se llevó a cabo mediante el software *Rayyan – Intelligent Systematic Review* (https://rayyan.ai/). Dos investigadores, de forma independiente, analizaron los títulos y los resúmenes; los estudios elegibles se leyeron en su totalidad. Las discrepancias fueron resueltas por un tercer investigador, y los duplicados se consideraron solo una vez.

La extracción de datos fue realizada por los dos investigadores mediante un instrumento estructurado en *Microsoft Excel* 2019^®^, resolviendo las discrepancias por consenso. El instrumento incluyó: identificación del estudio (título, autores, año, revista, país, idioma), aspectos metodológicos (diseño, tamaño de la muestra), principales resultados y conclusiones.

### Análisis y Tratamiento de los Datos

Los estudios se clasificaron según el nivel de evidencia del *Oxford Centre for Evidence-Based Medicine* (OCEBM), que varía desde revisiones sistemáticas de ensayos clínicos aleatorios (nivel 1a) hasta la opinión de expertos (nivel 5), y según la clasificación del JBI (niveles 1 a 4). Se constituyó un corpus textual con fragmentos de resúmenes, resultados y consideraciones finales (excluidas las referencias), reunidos en un único archivo en *Microsoft Word* y sometido a IRaMuTeQ.

El análisis léxico empleó la Clasificación Jerárquica Descendente (CJD) y el Análisis Factorial de Correspondencia (AFC), segmentando el texto en unidades de contexto y agrupando segmentos por similitud léxica y estadística. La interpretación y categorización de los términos siguió la Teoría de la Adaptación de Roy, organizando los datos en los modos fisiológico-funcional, autoconcepto-identidad, desempeño de roles e interdependencia.

Los resultados se presentaron mediante el diagrama de flujo *PRISMA-ScR* (identificación, selección, elegibilidad e inclusión), tablas con características metodológicas y hallazgos principales, cuadros con un resumen del nivel de evidencia y estrategias de búsqueda, además de figuras generadas por IRaMuTeQ (dendrograma de la CJD y mapa factorial de la AFC).

### Aspectos Éticos

Al tratarse de una revisión bibliográfica con datos secundarios de dominio público, el estudio no requiere la aprobación de un Comité de Ética en Investigación, respetando los derechos de autor y la fiabilidad de las referencias utilizadas.

## RESULTADOS

El proceso de revisión se inició con la identificación de 10 879 registros en diferentes bases de datos y fuentes manuales. La mayor parte de estos registros se obtuvo en PubMed (n = 10 006), seguida de Web of Science (n = 350), SciELO (n = 255), Portal de Periódicos CAPES (n = 133), Cochrane (n = 61), LILACS (n = 60) y RCAAP (n = 6). Además, se identificaron cuatro manuales/protocolos del Ministerio de Salud.

En la etapa de selección, se excluyeron 5.219 registros por no cumplir con los criterios de elegibilidad, por duplicidad o por ser irrecuperables.

De este modo, se analizaron 5.656 estudios en cuanto al título y el resumen, lo que dio lugar a la exclusión de 5.406 registros por irrelevancia temática o por no responder a la pregunta guía.

Un total de 250 estudios pasaron a la evaluación completa de la elegibilidad. Tras la lectura del texto completo, se excluyeron 241 estudios. Paralelamente, de los cuatro informes/manuales evaluados mediante métodos complementarios, dos se consideraron no elegibles. Al final del proceso de selección, 11 estudios (nueve artículos y dos informes/manuales) se incluyeron en la muestra final de esta revisión (Figura 1).

**Figura 1:** Esquema del proceso de selección de los estudios. Natal, RN, Brasil, 2025.

El corpus textual final de la investigación estuvo compuesto por 11 documentos, presentados en el Cuadro 1, los cuales fueron procesados y segmentados en 641 segmentos de texto (ST). Todos los segmentos se consideraron válidos para el análisis léxico, lo que dio como resultado un aprovechamiento total (100 %).

**Cuadro 1:** Estudos selecionados – Natal, RN, Brasil, 2025.

Estudio	País (Año)	Tipo de publicación/Tipo de estudio/Nivel de Evidencia
E1^(12)^	Brasil (2020)	Artículo/Cualitativo, descriptivo/Nivel de evidencia 4
E2^(13)^	Brasil (2019)	Artículo/Cuantitativo, descriptivo y exploratorio/Nivel de evidencia 4
E3^(14)^	Brasil (2019)	Artículo/Cuantitativo, descriptivo y exploratorio/Nivel de evidencia 4
E4^(15)^	Brasil (2022)	Artículo/Cualitativo, descriptivo y exploratorio/Nivel de evidencia 4
E5^(16)^	Brasil (2021)	Artículo/Cualitativo, descriptivo/Nivel de evidencia 4
E6^(17)^	Mozambique (2000)	Artículo/Cualitativo, descriptivo/Nivel de evidencia 2c
E7^(18)^	Brasil (2020)	Artículo/Cualitativo, investigación-acción/Nivel de evidencia 2c
E8^(19)^	Estados Unidos (1992)	Artículo/Revisión narrativa descriptiva/Nivel de evidencia 5
E9^(20)^	Reino Unido (1998)	Artículo/Revisión narrativa descriptiva/Nivel de evidencia 5
E10^(21)^	Brasil (2007)	Manual técnico/N/A/No especificado
E11^(22)^	Brasil (2016)	Manual técnico/N/A/No especificado

Tras el análisis de los datos, estos se organizaron y presentaron en tablas. Para facilitar la comprensión y la visualización, los estudios se identificaron con la letra «E» (estudio), seguida de números arábigos secuenciales (1, 2, 3, … 11), lo que dio lugar a las codificaciones E1, E2, E3, … E11.

El análisis realizado por el software IRaMuTeQ permitió clasificar los segmentos en siete clases temáticas distintas. Para garantizar la uniformidad terminológica, las clases se denominaron en singular y se estandarizaron de la siguiente manera: Clase 1 – Política y Acceso (16,22 %); Clase 2 – Gestión y Servicio (13,51 %); Clase 3 – Red Comunitaria (13,51 %); Clase 4 – Acogida y vínculo (13,51 %); Clase 5 – Atención materno-infantil (16,22 %); Clase 6 – Detección temprana (13,51 %); y Clase 7 – Diagnóstico y tratamiento (13,51 %), tal como se describe en la Figura 2.

**Figura 2:** Diagrama de las clases que componen el Dendograma del corpus textual (en portugués) correspondiente a los manuscritos de la muestra. Natal, RN, Brasil, 2025.

Estas clases no operan de manera aislada; por el contrario, representan la trayectoria de la atención de enfermería, que se inicia en las condiciones estructurales (Clases 1 y 2), atraviesa la relación interpersonal (Clases 3 y 4) y culmina en la intervención clínica directa (Clases 6 y 7).

La relación entre las clases debe interpretarse de izquierda a derecha. Inicialmente, el corpus se dividió en la primera iteración, separando la Clase 1 del resto. En la segunda iteración, el subcorpus restante se subdividió en dos, dando origen a la Clase 2 y a otro subcorpus. En la tercera iteración, este último subcorpus se dividió nuevamente, generando la Clase 5 por un lado y un nuevo subcorpus que, en la cuarta iteración, dio origen a las Clases 3 y 4. La clasificación jerárquica descendente se interrumpió en ese punto, ya que las cinco clases presentaron estabilidad, es decir, estaban compuestas por segmentos de texto con un vocabulario internamente homogéneo.

El Análisis Factorial de Correspondencia (AFC) mapeó la distribución espacial de las clases (Figura 3). Las clases identificadas se articulan directamente con la práctica clínica de la enfermería, al traducir dimensiones esenciales de la atención a la gestante con sífilis.

**Figura 3:** Análisis factorial de correspondencias de las palabras (en portugués) más frecuentes en cada clase léxica, basado en una clasificación jerárquica. Natal, RN, Brasil, 2025.

Los hallazgos léxicos revelan que las políticas y la gestión (Clases 1 y 2) influyen en la organización de los servicios y en la garantía de acceso a la atención prenatal, mientras que la red comunitaria y el vínculo (Clases 3 y 4) refuerzan la importancia de la escucha cualificada y de la confianza para la adherencia al tratamiento y el rastreo de parejas. Por su parte, la atención materno-infantil y la detección temprana (Clases 5 y 6) evidencian la necesidad de acciones integradas que aseguren la prevención de la transmisión vertical. A su vez, el diagnóstico y el tratamiento (Clase 7) destacan la dimensión técnica que, cuando se disocia de las demás, puede fragmentar la asistencia.

De este modo, el análisis léxico fortalece el vínculo entre el discurso científico y la práctica clínica, ofreciendo recursos para que el enfermero alinee las intervenciones técnicas con las dimensiones relacionales y organizacionales de la atención.

En la Figura 4, las palabras extraídas del corpus se correlacionaron con los modos adaptativos de la Teoría de Roy. La visualización confirma que la atención a la gestante con sífilis es multidimensional: los términos técnicos se articulan con términos de apoyo social, lo que evidencia que la adaptación eficaz depende tanto de la competencia clínica del enfermero (diagnóstico y tratamiento) como de su capacidad para articular la red y brindar acogida.

**Figura 4:** Términos relacionados con la atención a las gestantes con sífilis a la luz del marco teórico de adaptación de Roy. Natal, RN, Brasil.

El análisis factorial de correspondencias (AFC) reveló la organización semántica y léxica de los términos más frecuentes en el corpus textual. Como se ilustra en el gráfico, las Clases 1 (roja) y 2 (gris) se encuentran agrupadas en el cuadrante superior izquierdo, lo que indica una fuerte correlación entre los términos que las componen. Esta agrupación, que se extiende mínimamente hacia el cuadrante inferior izquierdo, sugiere que los conceptos representados por estas clases son semánticamente centrales e interrelacionados en el contexto del estudio.

En el cuadrante superior derecho, se observa la proximidad entre las Clases 3 (verde), 4 (cian) y 5 (azul). Esta disposición espacial refleja una convergencia de significados, lo que evidencia que, aunque son clases distintas, comparten un campo léxico común. La Clase 6 (azul oscuro), también ubicada en este cuadrante, se adentra sutilmente hacia el cuadrante inferior derecho, lo que sugiere una relación, aunque más atenuada, con los conceptos predominantes en esa región del gráfico.

Por último, la Clase 7 (morado) se destaca por su posición aislada y concentrada en el cuadrante inferior derecho. Este aislamiento indica que los términos de esta clase poseen especificidad semántica y léxica, diferenciándose de los demás grupos y representando un dominio conceptual más particularizado en el ámbito de la atención a la gestante con sífilis.

Este análisis pone de manifiesto la complejidad y la estructura subyacente del discurso sobre el tema, revelando patrones de asociación entre términos y conceptos de forma clara y visual.

## DISCUSIÓN

El análisis léxico puso de manifiesto la construcción discursiva del fenómeno de la sífilis en el embarazo. A partir de la agrupación de los términos, se identificaron núcleos de sentido que expresan diferentes ejes conceptuales de la atención. Aunque la organización es resultado del procesamiento sistemático del software, la inferencia clínica se basó en la interpretación de los investigadores^(8)^.

Las siete clases se estandarizaron de acuerdo con los ejes semánticos que representan: Política y Acceso, Gestión y Servicio, Red Comunitaria, Acogida y Vínculo, Atención Materno-Infantil, Detección Temprana y Diagnóstico y Tratamiento. Cada clase reúne términos interrelacionados que, en conjunto, profundizan la comprensión sobre las barreras y estrategias en la prevención de la transmisión vertical.

El análisis se basó en la Teoría de la Adaptación de Callista Roy, que concibe al individuo como un sistema adaptativo que responde a estímulos internos y externos^(23)^. Desde esta perspectiva, los términos de las clases manifiestan procesos adaptativos y desafíos impuestos por la sífilis, reflejando tanto las demandas de atención como las estrategias de afrontamiento^(24)^. Corresponde al personal de enfermería reconocer estas necesidades para promover el equilibrio adaptativo, con el objetivo de lograr un embarazo seguro y la prevención de resultados adversos para la díada madre-feto^(3)^.

El AFC demostró una proximidad entre la Clase 1 — Política y Acceso (términos: «regionalización», «coordinación», «acceso»)— y la Clase 2 —Gestión y Servicio (términos: «recursos», «planificación», «organización»)—.

Dicha conexión indica que la formulación de políticas públicas es indisociable de la capacidad organizativa de los servicios. Datos recientes señalan que la prevalencia de la sífilis en el embarazo alcanzó el 1,02 % en 2019, con un aumento significativo en los años anteriores. Este escenario refleja fragilidades estructurales en la red de atención. La literatura confirma que las fallas en la planificación y la asignación de recursos dificultan la implementación de políticas, limitan la adherencia al tratamiento y comprometen la integralidad de la atención^(4)^. Así, la superposición conceptual entre las clases refuerza la necesidad de políticas más eficientes y articuladas, capaces de enfrentar las desigualdades y garantizar la salud materno-infantil^(1,2)^.

El análisis también reveló una fuerte conexión entre la Clase 3 —Red Comunitaria— y la Clase 4 —Acogida y Vínculo—. La proximidad de términos como «comunitario» y «red» con «escuchar» y «confianza» demuestra que la atención a la gestante con sífilis trasciende la dimensión clínica. La atención prenatal en la atención primaria es un momento privilegiado para la identificación, notificación y tratamiento de los casos^(25)^. En este contexto, la acogida y la escucha cualificada se convierten en herramientas centrales para la construcción del vínculo y la confianza, favoreciendo la adherencia de las gestantes y sus parejas al tratamiento^(6)^. La literatura también destaca la relevancia de los Agentes Comunitarios de Salud (ACS) en la Estrategia de Salud Familiar (ESF), cuya actuación contribuye a la detección temprana de las gestantes y a la educación en salud en el ámbito comunitario^(26)^.

Por lo tanto, la lucha contra la sífilis gestacional debe integrar dimensiones relacionales, comunitarias y sociales. Esta integración promueve la confianza, fortalece los vínculos y reduce las barreras de acceso a los servicios de salud^(1,2)^.

El AFC evidenció una superposición de la Clase 5 — Atención Materno-Infantil— con la Clase 6 —Detección Temprana—. En la Clase 5, términos como «madre» y «niño» resaltan el enfoque en la díada y la continuidad de la atención. El término «desarrollo» apunta a la relevancia del vínculo afectivo, que puede verse afectado por el diagnóstico, mientras que «orientación» asume un papel central en el empoderamiento de la gestante. Paralelamente, la Clase 6 reúne términos como «campaña» y «búsqueda», que reflejan el pilar de la salud pública: la acción proactiva. La «búsqueda activa» y la «detección temprana» refuerzan la necesidad de políticas ágiles, capaces de superar la espera pasiva del diagnóstico. La superposición de estas clases demuestra que la atención materno-infantil y la detección temprana son dimensiones interrelacionadas, que forman una estrategia única que depende del vínculo, la acogida y la proactividad de los servicios de salud^(27)^.

La Clase 7 —Diagnóstico y tratamiento— aparece en una posición aislada y concentrada, marcada por términos técnicos como «examen» y «prescripción». Esta separación no es solo gráfica, sino que refleja una disociación conceptual entre los procedimientos clínicos y el contexto social y humano de la gestante.

En resumen, los resultados sugieren que el éxito en el manejo de la sífilis gestacional va más allá de la mera implementación de protocolos. Es necesaria la articulación entre acciones de detección y tratamiento (biológicas), atención humanizada, prácticas de acogida y vínculo (psicosociales), además de una red integrada y servicios organizados y accesibles (gestión). Esta integración alinea las intervenciones técnicas con las dimensiones sociales y humanas de la atención, ofreciendo fundamentos para diagnósticos de enfermería más precisos e intervenciones efectivas en la atención prenatal.

## CONCLUSIÓN

El análisis léxico de los términos relacionados con la atención a la gestante con sífilis, a la luz de la literatura de enfermería, permitió identificar el vocabulario más recurrente sobre el fenómeno. Con la ayuda del software IRaMuTeQ, se evidenció un conjunto de términos fuertemente asociados al tema.

Los términos más relevantes fueron: políticas, acceso, gestión, servicios, red, comunitario, acogida, vínculo, atención, madre, niño, detección, campaña, búsqueda, diagnóstico, examen y prescripción.

Este estudio sobre el léxico de la sífilis gestacional puede contribuir a mejorar el razonamiento clínico y crítico del personal de enfermería. Al proporcionar respaldo científico, permite identificar desafíos y estrategias de atención, lo que ayuda a formular diagnósticos de enfermería más precisos y a aplicar intervenciones que tengan en cuenta las dimensiones clínicas, sociales y humanas de la gestante.

Se recomienda que futuras investigaciones profundicen en el análisis de la interrelación entre estos términos, con el fin de validar y ampliar los resultados obtenidos.

## DISPONIBILIDAD DE DATOS

El conjunto de datos que respaldan las conclusiones de este estudio está disponible previa solicitud al autor correspondiente.
